# Repeated small-volume exchange transfusion for hyperleukocytosis in pediatric acute leukemia: A retrospective analysis

**DOI:** 10.3389/fped.2023.1155481

**Published:** 2023-03-22

**Authors:** Dongxiu Zhang, Hailong Lin, Leting Huang

**Affiliations:** ^1^ Department of Pediatrics, the Second Affiliated Hospital and Yuying Children's Hospital, Wenzhou Medical University, Wenzhou, China; ^2^ Department of Hematology and Oncology, the Second Affiliated Hospital and Yuying Children's Hospital, Wenzhou Medical University, Wenzhou, China

**Keywords:** leukemia, hyperleukocytosis, leukapheresis, exchange transfusion, leukostasis

## Abstract

**Introduction:**

Leukapheresis reduces hyperleukocytosis in children with acute leukemia. Although the usefulness of this procedure is under debate, a repeated small-volume exchange transfusion along with leukapheresis yielded satisfactory results.

**Methods:**

Forty-seven patients with acute leukemia [32 acute lymphocytic leukemia (ALL) and 15 acute myeloblastic leukemia (AML)] were enrolled between January 2017 and June 2022 and underwent repeated small-volume exchange transfusion. The following were measured: demographic and clinical characteristics, time of the procedure, PWBC (peripheral white blood cell) count, hemoglobin, platelet count, blood biochemistry, electrolytes, coagulation, leukostasis, TLS (tumor lysis syndrome), DIC (disseminated intravascular coagulopathy), adverse events (AEs), and serious AEs (SAEs).

**Results:**

The demographic and clinical characteristics were not significantly different between ALL and AML patients, but differences were observed in PWBC counts (424.2 ± 135.6 vs. 223.8 ± 58.0 × 109/L). The procedures needed 3–8 processes, and the average procedure time was not significantly different between ALL and AML. The PWBC count gradually reduced to <100 × 109/L; hemoglobin, platelet count, K+, Na+, and Ca2+ were unchanged. Alanine aminotransferase, aspartate aminotransferase, total bilirubin, blood urea nitrogen, creatinine, troponin-I, creatine kinase-MB, prothrombin time, and activated partial thromboplastin time maintained normal or recovered from abnormal ranges. The manifestations of leukostasis, TLS, and DIC improved or disappeared. No AEs and SAEs occurred. The required total blood volume was based on initial PWBC count, manifestations of leukostasis, and age.

**Conclusions:**

Our finding suggests that repeated small-volume exchange transfusion is effective and safe for treating hyperleukocytosis in children with acute leukemia.

## 1. Introduction

Hyperleukocytosis is defined as a white blood cell (WBC) count of >100 × 10^9^/L and is often associated with early mortality in patients with leukemic processes. Hyperleukocytosis can induce leukostasis, tumor lysis syndrome (TLS), and disseminated intravascular coagulopathy (DIC) (1, 2). Leukapheresis is a laboratory procedure in which WBCs are separated from the blood. The leukocytes are mechanically removed, and the remaining blood is returned to the patient (3). However, the blood volume required for this procedure should be two to four times the patient's blood volume, which predisposes to complications (4). In this study, a repeated small-volume exchange transfusion was conducted along with leukapheresis to treat hyperleukocytosis in children with acute leukemia. The results were satisfactory, and the procedure did not require mechanical separation.

## 2. Methods

The present study retrospectively analyzed the medical records of hospitalized patients with acute leukemia admitted to The Second Affiliated Hospital and Yuying Children's Hospital of Wenzhou Medical University, Zhejiang Province, China, between January 2017 and June 2022. This study was approved by the Institutional Ethics Committee of The Second Affiliated Hospital and Yuying Children's Hospital of Wenzhou Medical University (approval number: 1-2022-12). The inclusion criteria were as follows: (1) confirmed acute leukemia based on the 2016 revision of the WHO classification of hematopoietic and lymphoid tumors, with acute leukemia in the primary bone marrow cells (5); (2) hyperleukocytosis with peripheral WBC (PWBC) counts >100 × 10^9^/L (6); and (3) classification of leukemia as acute lymphocytic leukemia (ALL) or acute myeloblastic leukemia (AML). The exclusion criteria were as follows: (1) leukemoid reaction (response to stress or infection) and (2) use of glucocorticoids before and/or within 3 days.

The repeated small-volume exchange transfusion did not require specialized equipment for separation. Regarding the procedure, we first established intravenous routes using the peripheral and/or central venous catheter. Thereafter, a total blood volume of 10–15 ml/kg was extracted from the venous catheter per day, and the blood was not reinfused. The same volume of packed red blood cells was then infused from the intravenous route, and the procedure was performed daily until a PWBC count of <100 × 10^9^/L was attained.

PWBC count, hemoglobin, and platelet count for each procedure were measured twice daily (before and after the procedure). Indicators of serum biochemistry, electrolytes, and coagulation were evaluated three times every 2–3 days. Serum biochemistry was assessed, including alanine aminotransferase (ALT, standard value: 9–50 IU/L), aspartate aminotransferase (AST, standard value: 15–40 IU/L), total bilirubin (TBil, standard value: 6.8–34.2 μmol/L), blood urea nitrogen (BUN, standard value: 2.8–8.2 mmol/L), creatinine (standard value: 53.0–106.0 μmol/L), troponin-I (standard value: 0.0–3.4 ng/L), and creatine kinase-MB (CK-MB, standard value: 0.0–5.0 μg/L). Electrolytes were also assessed, including K^+^ (standard value: 3.5–5.5 mmol/L), Na^+^ (standard value: 135.0–145.0 mmol/L), and Ca^2+^ (standard value: 2.0–2.5 mmol/L). Coagulation, including prothrombin time (PT, standard value: 11.0–14.0 s) and activated partial thromboplastin time (APTT, standard value: 27.0–40.0 s), was also assessed. Vital signs, including body temperature, respiration, heart rate, and blood pressure, were monitored throughout the procedure.

Some conditions were corrected before the procedure, such as hemoglobin <70 g/L, platelet count <20 × 10^9^/L, and DIC. Hydration (0.45% sodium chloride IV at 3 L/m/day or 200 mL/kg/day if the child weighed ≤10 kg), and anti-infection treatments were necessary in most cases. There was no alkalinization treatment.

If immature cells existed in the peripheral blood before bone marrow biopsy, repeated small-volume exchange transfusion was started immediately, as such cells suggested acute leukemia. When the type of acute leukemia was confirmed (approximately 3–4 days after bone marrow aspiration), a low-dose cytoreduction strategy was performed along with exchange transfusion to reduce hyperleukocytosis. The low-dose cytoreduction strategy was 5 mg/m^2^ of prednisone daily for ALL (7) and 100 mg/m^2^ of cytarabine daily for AML (8). The exchange transfusion was completed when PWBC <100 × 10^9^/L. However, the low-dose cytoreduction strategy was continued until PWBC <50 × 10^9^/L in ALL and <30 × 10^9^/L in AML.

**Figure 1:**
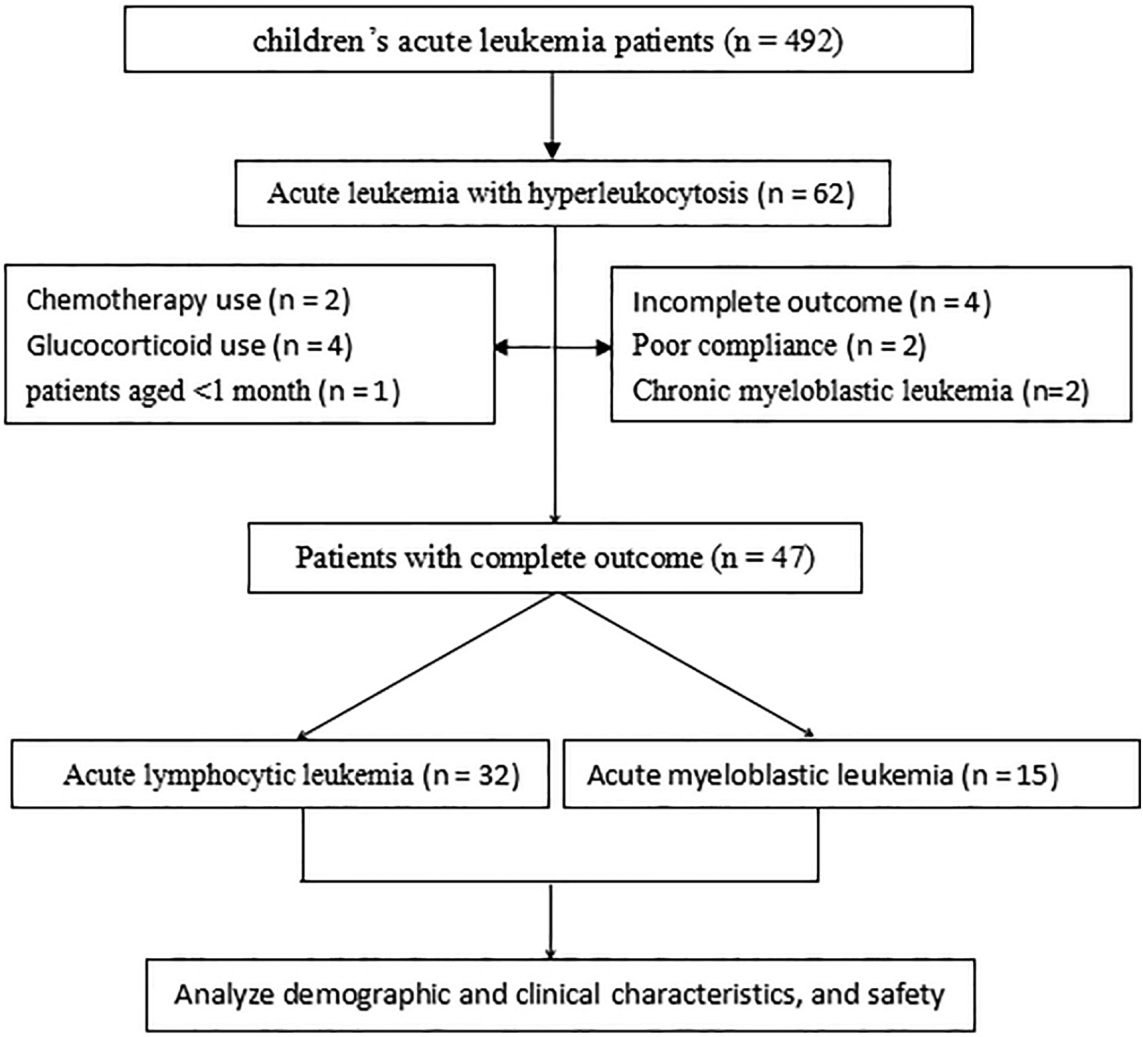
Flowchart of patients included in the investigation.

Data retrieved from the medical records included the demographic and clinical characteristics of the patients, medication outcomes, and treatment safety. Patient records/information in the database were anonymized and de-identified before analysis (Figure 1).

### 2.1. Analysis

Data processing and statistical analyses were performed using the Statistical Package for the Social Sciences (SPSS) version 17.0. The results were compared using the t-test or chi-square test (including Fisher's exact test), analysis of variance (ANOVA), and logistic regression.

## 3. Results

### 3.1. Demographic and clinical characteristics

Forty-seven patients were enrolled in the present study, including 32 ALL and 15 AML patients. The majority of the patients were male (55.3%), and the mean patient age was 72.4 ± 34.9 months. Of these, 61.7% were from urban areas, and 6.5% had a family history of leukemia. Almost all patients exhibited symptoms, including fever, bleeding, and anemia. Among the patients, 83.0%, 9.5%, and 7.1% had leukostasis, TLS, and DIC, respectively. No significant differences were observed between the ALL and AML patients in symptoms, sex, age, location of residence, and family history of leukemia (all *P* > 0.05). The mean PWBC count for the ALL and AML groups was 424.2 ± 135.6 × 10^9^/L and 223.8 ± 58.0 × 10^9^/L, respectively, with significant differences between them (*P* < 0.05). Furthermore, platelet count, hemoglobin, PT, and APTT for ALL and AML were 33.6 ± 12.5 × 10^9^/L vs. 27.4 ± 9.1 × 10^9^/L, 62.4 ± 11.7 g/L vs. 57.0 ± 12.3 g/L, 15.2 ± 2.9 s vs. 15.6 ± 2.7 s, and 44.7 ± 9.4 vs. 45.8 ± 9.6 s, respectively, with no significant differences (all *P* > 0.05; Table 1).

**Table 1:** Demographic and clinical characteristics of cases.

Variable	ALL	AML	*χ*2/*t*-test (*P*-value)
Sex, No. (%)			
Male	17 (57.8)	9 (60.0)	0.1953 (0.6585)
Female	15 (42.2)	6 (40.0)	
Age (months)	65.8 ± 32.3	88.4 ± 37.9	2.1155 (0.0400)
Location of residence, No. (%)			
Urban	21 (65.6)	8 (53.3)	0.6530 (0.4191)
Rural	11 (34.4)	7 (46.7)	
Family history of leukemia, No. (%)			
Yes	2 (6.3)	1 (6.7)	0.0030 (0.9566)
No	30 (93.7)	14 (93.3)	
Symptoms, No. (%)			
Fever^a^	32 (100.0)	15 (100.0)	— (1.0000)
Bleeding^a^	27 (84.4)	15 (100.0)	— (0.1617)
Anemia^a^	32 (100.0)	15 (100.0)	— (1.0000)
Leukostasis	25 (78.1)	14 (93.3)	0.7690 (0.3805)
TLS	2 (6.3)	2 (13.3)	0.6581 (0.4172)
DIC	2 (6.3)	1 (6.7)	0.0030 (0.9566)
PWBC count ( × 109/L)	424.2 ± 135.6	223.8 ± 58.0	5.4689 (0.0000)
Platelet count ( × 109/L)	33.6 ± 12.5	27.4 ± 9.1	1.8868 (0.2080)
Hemoglobin (g/L)	62.4 ± 11.7	57.0 ± 12.3	1.4514 (0.1536)
PT (s)	15.2 ± 2.9	15.6 ± 2.7	0.4502 (0.6547)
APTT (s)	44.7 ± 9.4	45.8 ± 9.6	0.3715 (0.7120)

ALL, acute lymphocytic leukemia; AML, acute myeloblastic leukemia; TLS, tumor lysis syndrome; DIC, disseminated intravascular coagulopathy; PWBC, peripheral white blood cell; PT, prothrombin time; APTT, activated partial thromboplastin time.

Data are presented as *n* (%) or the mean ± standard deviation.

^a^ The data for the clinical manifestations were compared using Fisher's exact test.

### 3.2. Clinical manifestations

Small-volume exchange transfusion was repeated 3–8 times in patients, with an average of 5.03 ± 1.50 procedures [5.28 ± 1.44 for ALL and 4.67 ± 1.63 for AML, there were no significant differences between them (*t* = 1.30, *P* = 0.20)]. When converted to blood volume, the total volume was exchanged 0.52 ± 0.16 times.

**Figure 2:**
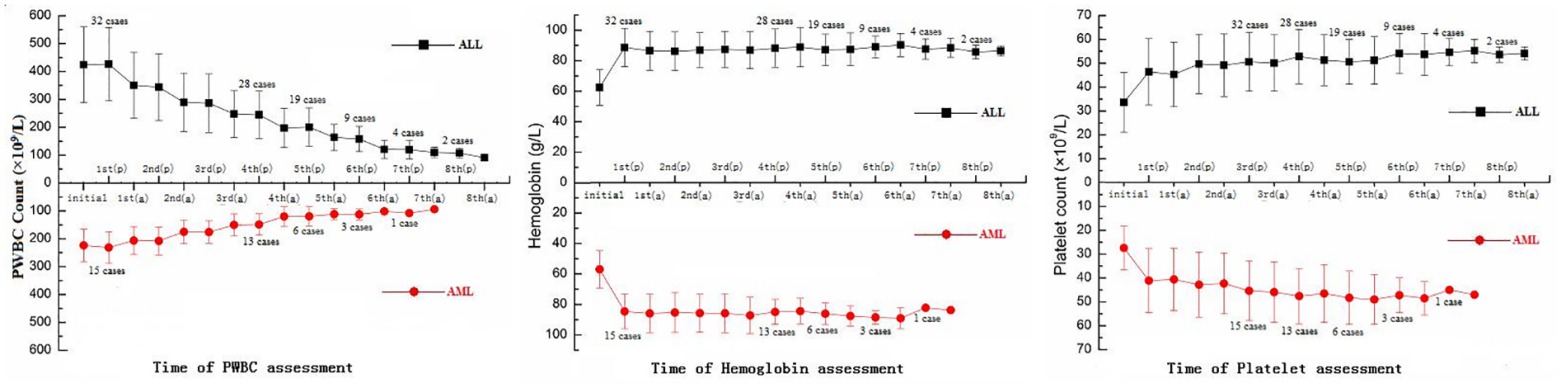
The total number of 47 patients with PWBC count, hemoglobin, and platelet count, including 32 ALL and 15 AML patients. (p), prior to the process; (a), after the process; PWBC, peripheral white blood cell; ALL, acute lymphocytic leukemia; AML, acute myeloblastic leukemia.

PWBC count, platelet count, and hemoglobin were measured throughout the procedure. The initial test result was corrected before the procedure when hemoglobin <70 g/L and/or platelet count <20 × 10^9^/L. Furthermore, from the first day of the procedure, all the indicaors for two detections (before and after a process per day). The PWBC count in 42 cases gradually reduced to <100 × 10^9^/L, with 3–8 processes in ALL and 3–7 in AML. Nineteen of the 47 patients (12 in ALL and 7 in AML) completed the procedure, with 3–4 processes before the low-dose cytoreduction strategy. A normal hemoglobin level was maintained during the entire procedure in all cases, and the same amount of packed red blood cells infused after 10–15 ml/kg of whole blood was extracted for bloodletting. At the final detection, the platelet count was maintained and increased in 28 patients. There were no cases of packed platelets infusion (Figure 2).

### 3.3. Detection of blood biochemistry, electrolytes, and coagulation

Blood biochemistry of the patients, including ALT, AST, TBil, BUN, creatinine, troponin-I, and CK-MB, tended to improve with significant differences between times of measurement of ALT (*F *= 5.58, *P* = 0.0081), AST (*F *= 6.22, *P* = 0.0092), TBil (*F *= 11.06, *P* = 0.0011), BUN (*F *= 16.21, *P* = 0.0003), creatinine (*F *= 14.51, *P* = 0.0022), and CK-MB (*F* = 5.19, *P* = 0.0139), but troponin-I showed no significant difference (*F *= 2.45, *P* = 0.1427). Coagulation also tended to improve with significant differences between times of PT (*F *= 4.26, *P* = 0.0183) and APTT (*F *= 9.82, *P* = 0.0021). There were no significant differences between the times of electrolyte detection for Na^+^ (*F* = 1.88, *P* = 0.8310) and Ca^2+^ (*F* = 1.89, *P* = 0.1754), but K^+^ (*F* = 16.29, *P* = 0.0002) showed significant differences (Table 2).

**Table 2:** Three assessments of blood biochemistry, electrolytes, and coagulation function in ALL and AML patients.

Variable	First	Second	Third	*F* (*P*-value)
ALT (IU/L)				
ALL	74.5 ± 37.4	63.5 ± 26.7	48.8 ± 22.1	6.14 (0.0031)
AML	79.5 ± 40.2	68.6 ± 28.8	46.7 ± 20.6	4.38 (0.0188)
AST (IU/L)				
ALL	79.4 ± 65.9	53.8 ± 35.3	36.4 ± 17.6	7.61 (0.0009)
AML	75.2 ± 62.1	55.0 ± 37.9	35.3 ± 14.1	3.26 (0.0482)
TBil (μmol/L)				
ALL	35.0 ± 15.9	29.2 ± 7.7	21.3 ± 6.0	13.22 (0.0000)
AML	37.8 ± 17.2	28.4 ± 8.7	22.6 ± 6.3	6.44 (0.0036)
BUN (mmol/L)				
ALL	8.54 ± 2.43	7.12 ± 2.19	5.33 ± 1.32	19.96 (0.0000)
AML	8.40 ± 2.24	6.86 ± 2.06	5.51 ± 1.49	8.20 (0.0010)
Creatinine (μmol/L)				
ALL	128.9 ± 44.2	107.6 ± 32.0	78.3 ± 18.4	18.68 (0.0000)
AML	124.3 ± 45.8	100.7 ± 31.8	82.7 ± 19.7	5.60 (0.0070)
Troponin-I (ng/L)				
ALL	2.37 ± 1.64	1.85 ± 1.06	1.62 ± 0.89	3.08 (0.0508)
AML	2.28 ± 1.56	1.92 ± 1.13	1.61 ± 0.92	1.11 (0.3389)
CK-MB (μg/L)				
ALL	3.42 ± 2.88	2.23 ± 1.62	1.78 ± 0.85	5.92 (0.0038)
AML	3.57 ± 2.60	2.58 ± 1.70	1.75 ± 0.82	3.62 (0.0355)
Na^+^ (mmol/L)				
ALL	139.7 ± 3.1	140.1 ± 2.5	139.9 ± 2.7	0.17 (0.8474)
AML	139.3 ± 3.3	139.8 ± 2.8	140.0 ± 2.6	0.23 (0.7959)
K^+^ (mmol/L)				
ALL	5.44 ± 0.71	4.76 ± 0.57	4.42 ± 0.60	21.78 (0.0000)
AML	5.38 ± 0.66	4.68 ± 0.59	4.47 ± 0.63	8.65 (0.0007)
Ca^2+^ (mmol/L)				
ALL	2.01 ± 0.15	1.96 ± 0.11	1.95 ± 0.12	2.02 (0.1378)
AML	1.99 ± 0.14	1.92 ± 0.12	1.93 ± 0.10	1.47 (0.2424)
PT (s)				
ALL	13.8 ± 2.5	13.3 ± 1.7	12.5 ± 0.9	4.15 (0.0188)
AML	14.6 ± 2.3	13.6 ± 1.6	12.7 ± 1.1	4.49 (0.0171)
APTT (s)				
ALL	40.4 ± 8.3	36.1 ± 5.1	33.3 ± 3.1	11.75 (0.0000)
AML	42.8 ± 9.2	37.3 ± 6.4	34.6 ± 3.5	5.70 (0.0065)

ALL, acute lymphocytic leukemia; AML, acute myeloblastic leukemia; ALT, alanine aminotransferase; AST, aspartate aminotransferase; TBil, total bilirubin; BUN, blood urea nitrogen; CK-MB, creatine kinase; PT, prothrombin time; APTT, activated partial thromboplastin time; ANOVA, analysis of variance.

The results were compared using ANOVA.

### 3.4. Leukostasis, TLS, and DIC

Thirty-nine (83.0%), four (9.5%), and three (7.1%) patients manifested leukostasis, TLS, and DIC, respectively, before the procedure. The clinical manifestations of leukostasis included headache (32 cases), blurred vision (4 cases), transient ischemic attacks (5 cases), and dyspnea (19 cases), but the manifestations disappeared after the leukapheresis procedure. DIC patients were treated with packed platelet suspension, fresh frozen plasma, and/or prothrombin complex once before the procedure. In the entire procedure, the platelet count was maintained at >30 × 10^9^/L, and the PT and APTT gradually normalized. Manifestations of TLS, such as high blood potassium (hyperkalemia), low blood calcium (hypocalcemia), and high blood uric acid (hyperuricemia), recovered to normal, although a low-dose cytoreduction strategy was performed.

### 3.5. Adverse events and serious adverse events

No adverse events (AEs) or serious AEs (SAEs) were observed, including blood pressure instability, organ disorder, and transfusion reaction. Organ indicators, such as ALT, AST, TBil, BUN, creatinine, troponin-I, CK-MB, PT, and APTT, recovered to normal or maintained stability; deterioration was not observed. Infection during blood transfusion was also a concern for the patients; therefore, the patients were tested for HIV, *Treponema pallidum*, hepatitis B virus, and hepatitis C virus twice with antigen and/or antibody. In addition, epstein-barr virus (EBV)-DNA and Human cytomegaloviru (HCMV)-DNA were also checked twice (before and after the procedure). In this study, no patient was infected by the above pathogens before or after the procedure. Catheter-related bacterial infection was also not detected with intraductal blood for hemoculture in the procedure.

### 3.6. Logistic regression analysis

Although all patients achieved a PWBC count of <100 × 10^9^/L, the procedure time varied. This means that each patient received a different blood volume for exchange transfusion. Twenty-one patients completed the procedure 3–4 times before the low-dose cytoreduction strategy (no cytoreduction cases), and 26 patients received the low-dose cytoreduction strategy during the procedure (cytoreduction cases). The present study analyzed 26 variables between the patients to see whether they received the low-dose cytoreduction strategy. These variables included the type of acute leukemia, demographic characteristics (sex, age, location of residence, and family history of leukemia), symptoms (bleeding, anemia, leukostasis, TLS, and DIC), initial detection of PWBC count, platelet count, hemoglobin, blood biochemistry (ALT, AST, TBil, BUN, creatinine, troponin-I, and CK-MB), electrolytes (K^+^, Na^+^, and Ca^2+^), coagulation (PT and APTT), and cooperative treatment (whether or not antibiotics were used). Logistic regression was performed to compare the variables, and an R^2^ value of 0.905 was obtained for the model. The predictive accuracy of the model was 96.2%. The analysis also demonstrated that the initial PWBC count, leukostasis, and age were significant predictors of the required total blood volume (*P* = 0.001, 0.008, and 0.012, respectively), indicating their effect on procedure times. The odds ratios (95% CIs) for these three variables were 1.483 (1.169–1.761), 0.677 (0.534–0.851), and 0.631 (0.421–0.867), respectively (Table 3).

**Table 3:** Logistic regression analysis of the no cytoreduction and cytoreduction cases.

Variable	No cytoreduction	Cytoreduction	*P*-value	Unadjusted OR (95% CI)	Adjusted OR (95% CI)
PWBC (×10^9^/L)	206.7 ± 8.3	486.7 ± 103.7	0.001	1.483 (1.169–1.761)	1.497 (1.176–1.851)
Leukostasis, No. (%)	14 (66.7)	25 (96.2)	0.008	0.672 (0.534–0.851)	0.686 (0.546–0.867)
Age (months)	60.7 ± 26.8	92.2 ± 34.1	0.012	0.631 (0.421–0.817)	0.654 (0.475–0.842)

PWBC, peripheral white blood cells; 95% CI, 95% confidence interval; OR, odds ratio.

There were 47 patients, of which 21 were no cytoreduction cases and 26 were cytoreduction cases.

## 4. Discussion

Hyperleukocytosis is defined as an initial PWBC count of >100 × 10^9^/L at presentation or relapse in children with ALL and AML (1). The incidence of hyperleukocytosis ranges from 12% to 25% and 10% to 30% in children with AML and ALL, respectively (9). Leukostasis, DIC, and TLS are the three main clinical manifestations of hyperleukocytosis, which can cause life-threatening complications in patients (2, 3). Thus, reducing the PWBC count is critical for hyperleukocytosis to limit leukostasis, DIC, and TLS and prevent early death (3, 10). There are two ways to rapidly reduce the PWBC count in patients diagnosed with acute leukemia: early start of chemotherapy and leukapheresis, although the relative merits and risks of both methods have not been conclusively proven (1, 11).

Conventional therapeutic leukapheresis is the separation and removal of abnormal leukocytes from whole blood while the remainder is returned to the patient (3, 12). This process requires double the blood volume and is expected to reduce the initial PWBC by 50% (13). The total blood volume for one process is calculated as 100 mL/kg for infants <1 month of age, 80 mL/kg for children aged 1 month–10 years, and 70 mL/kg for children >10 years (14). In apheresis devices (blood cell separators), WBCs and their precursors are separated from patients’ blood by centrifugation (15, 16). However, there are three main reasons why the use of leukapheresis for hyperleukocytosis is still debated. First, the effect is generally transient since PWBC counts often rebound quickly after leukapheresis (13, 17). Second, although leukapheresis is generally well-tolerated, hypercalcemia, the use of anticoagulants, aggravation of preexisting thrombopenia, and the process itself may put patients at risk in addition to their unstable condition, such as cardiovascular comorbidities, hemodynamic instability, and coagulation disturbances (18, 19). Finally, infrastructural requirements, trained personnel, and costs linked to leukapheresis have to be considered (20).

An exchange transfusion is often practical when hyperleukocytosis is complicated with severe anemia and may contribute to rapid leukocyte reduction with acceptable risk (21, 22). Strauss et al. found that manual exchange transfusion without hemapheresis instrumentation was efficacious for two patients with acute leukemia (a neonate with AML and a 2.5-year-old with acute promyelocytic leukemia) (23). Nelson et al. assumed that patients with ALL who present without the life-threatening complications of an extremely high leukocyte count could be safely and effectively managed with intravenous hydration, alkalinization, and allopurinol therapy exchange transfusion and leukapheresis (24). A study reported that leukapheresis or exchange transfusion was used in 24% of patients (a total of 890 patients) with hyperleukocytosis without impact on survival (25). Therefore, the effect of leukapheresis or exchange transfusion on short- and long-term outcomes in pediatric patients with acute leukemia and initial hyperleukocytosis should be evaluated in future multicenter studies or clinical registries (22).

If the apheresis equipment is unavailable, despite being the first choice, judicious phlebotomies with concurrent blood and/or plasma replacement may be used (26). This study presents a leukapheresis method without the need for equipment—repeated small-volume exchange transfusion for leukapheresis treatment of hyperleukocytosis in children with acute leukemia. Leukapheresis needs 70–100 ml/kg total blood volume for transfusion at one time. However, it is challenging to conduct the treatment without apheresis equipment because of the potential for discontinued transfusion, blood pressure instability, and large volume of blood and/or plasma required for replacement. Repeated small-volume exchange transfusion requires 3–8 processes, with 10–15 mL/kg of whole blood extracted from the venous catheter daily (approximately 0.52 ± 0.16 times the total blood volume was exchanged in individuals). This means some patients do not require a 70–100 mL/kg total blood volume for transfusion to achieve PWBC of <100 × 10^9^/L, and the manifestations of leukostasis, TLS, and DIC were alleviated or disappeared after leukapheresis.

There are no evidence-based guidelines regarding when to start leukapheresis. It is typically reserved for patients with symptomatic hyperleukocytosis who cannot start induction chemotherapy immediately (27). In most centers, leukapheresis is initiated in AML with PWBC >100 × 10^9^/L, while it is performed in ALL with PWBC of >300 × 10^9^/L (28). However, others consider the start time to be PWBC >200 × 10^9^/L and >400 × 10^9^/L for AML and ALL, respectively (29). It is debatable whether the above views are appropriate because all are based on a 70–100 mL/kg total blood volume for a single exchange transfusion. In this study, 21 patients finished the procedure after only 3–4 processes of repeated small-volume exchange transfusion without low-dose cytoreduction strategy; they attained PWBC <100 × 10^9^/L, and the manifestations of leukostasis, TLS, and DIC were alleviated or even disappeared. Regardless of AML or ALL, the manifestations of leukostasis, TLS, and DIC perhaps occurred when PWBC >100 × 10^9^/L. If the initial leukapheresis procedure starts on time, patients will not require a 70–100 mL/kg total blood volume for exchange transfusion if methods like repeated small-volume exchange transfusion are performed. Twenty-six patients needed 4–8 processes along with the low-dose cytoreduction strategy. They all completed the procedures without events, and some probably needed a total blood volume for exchange transfusion exceeding 70–100 mL/kg. The required total blood volume was mainly based on initial PWBC counts, manifestations of leukostasis, and age based on the logistic regression.

The efficiency of leukapheresis is the main reason exchange transfusion is applied, and PWBC count is the primary indicator of efficiency. In this study, the PWBC counts of all patients gradually declined to <100 × 10^9^/L over 3–8 processes, steadily declining daily by a PWBC count of 8%–20%. Moreover, no patient had a rapid increase in PWBC counts during and even after the procedure, perhaps due to the daily processes and low-dose cytoreduction strategy performed immediately when the type of acute leukemia was confirmed. Whether repeated small-volume exchange transfusion or low-dose cytoreduction strategy contributed more to leukapheresis in the cytoreduction cases is unknown, and evidence, such as randomized controlled trials, has not verified this (30). The manifestations of leukostasis, TLS, and DIC are also indicators of efficiency. In patients who underwent the procedure, manifestations alleviated or even disappeared, and PWBC counts declined. A study found that patients with WBC >200,000/μL had slightly better outcomes with exchange transfusion compared to those without exchange transfusion (early death rate 7.5% vs. 21.2%, *P* = 0.055) (31). Another study showed that manual whole blood exchange transfusion was favored over automated leukapheresis in infants <10 kg because of the technical and complication risks associated with acute leukemia (AL) (32). However, it was challenging to assess whether leukapheresis can reduce the rate of early mortality in this study because it was conducted with a low-dose cytoreduction strategy, and induction chemotherapy treatment was started soon after leukapheresis. However, no death was observed before chemotherapy induction in the study.

Safety is also a concern before starting leukapheresis. Some studies observed that patients might lose platelets when equipment is used (33, 34). However, in repeated small-volume exchange transfusion, the platelet counts were maintained, and some even recovered compared to the first platelet count. Blood biochemistry (ALT, AST, TBil, BUN, creatinine, troponin-I, and CK-MB) and coagulation (PT and APTT) indices also maintained normal levels and recovered from abnormal values. No patients appeared to deteriorate concerning the above indicators. Hemoglobin and Na^+^ were maintained because the packed red blood cell suspension and hydration treatment were applied throughout the procedure. Such indicators make it difficult to assess the impact of repeated small-volume exchange transfusion. Hyperleukocytosis usually accompanies hyperkalemia (35), so most patients showed high K^+^ levels, but as the procedure progressed, the situation gradually improved. Although some patients had hypocalcemia (35), and Ca^2+^ appeared decreased in some patients, convulsion did not occur due to hypocalcemia. Blood pressure instability and transfusion reaction were unnoted in this study, possibly due to the adequate preoperative preparation and small volume for exchange transfusion.

There are two problems with repeated small-volume exchange transfusion. First, the transfusion of packed red blood was needed daily for 3–8 processes to perform leukapheresis without equipment. However, it was a small daily volume. Venous access was another problem due to the different processes and different ages; some patients only needed peripheral venous access, while some needed a central venous catheter and even additional peripheral venous access, which was difficult to assess accurately.

Finally, the reason the study was not a randomized controlled trial, unlike leukapheresis with equipment, is because of failure (invalid or unstable condition) in six patients before the repeated small-volume exchange transfusion. However, although our study was retrospective, its results are plausible.

## 5. Conclusions

Repeated small-volume exchange transfusion as a leukapheresis procedure without equipment was suitable for treating hyperleukocytosis in children with acute leukemia (ALL or AML), as evidenced by its moderate attenuation of PWBC counts and improvement of leukostasis, TLS, and DIC manifestations in patients with hyperleukocytosis. In addition, repeated small-volume exchange transfusion was deemed safe.

## Data Availability

The original contributions presented in the study are included in the article/**Supplementary Material**, further inquiries can be directed to the corresponding author.
